# Adults in private family law proceedings in Wales: characteristics and vulnerabilities.

**DOI:** 10.23889/ijpds.v7i3.1910

**Published:** 2022-08-25

**Authors:** Linda Cusworth, Claire Hargreaves, Bachar Alrouh, Karen Broadhurst, Lucy Griffiths, Ashley Akbari, Ann John, Rhodri Johnson

**Affiliations:** 1 Centre for Child and Family Justice Research, Lancaster University; 2 Swansea University

## Objectives

Private law children cases are disputes, usually between parents after relationship breakdown, about arrangements for a child’s upbringing, such as where they should live and/or who they should see. To inform policy and practice, more information is needed about the families involved, including their characteristics, circumstances and vulnerabilities.

## Approach

This paper presents findings from research by the Family Justice Data Partnership – a collaboration between Lancaster and Swansea Universities – funded by the Nuffield Family Justice Observatory. Anonymised routinely-collected, individual-level, population-scale family justice data from Cafcass Cymru was linked with hospital and GP records, within the Secure Anonymised Information Linkage (SAIL) Databank for 18,653 adults involved in private law proceedings in Wales between 2014/15 and 2019/20 and a comparison group of 186,470 adults not involved in court proceedings, matched on age, gender, deprivation. The proportion of adults in the two groups with several health-related vulnerabilities recorded was compared.

## Results

Adults involved in private law proceedings were found to have higher prevalence of mental health problems, substance use, self-harm and exposure to domestic violence and abuse than the matched comparison group. Both men and women involved in private law proceedings were between two and three times as likely to have anxiety and depression than their peers in the comparison group. Almost 1 in 6 (15.3%) cohort women and more than 1 in 10 cohort men (10.7%) had a history of self-harm, again indicating a level of vulnerability more than double that observed in the comparison group. The research also revealed greater overall use of healthcare services by adults in private law proceedings, with the largest differences seen in the need for emergency health care.

## Conclusion

This research has uncovered the heightened needs and vulnerabilities of both women and men involved in private family law applications. The findings have important implications for the family justice system, and for health and other services, and need to be considered in relation to the current programme of reform.

